# Supplementary material for: Revisiting the van der Waals Epitaxy in the Case of (Bi0.4Sb0.6)2Te3 Thin Films on Dissimilar Substrates
Source: Nanomaterials (Basel). 2022 May 24;12(11):1790. doi: 10.3390/nano12111790 (PMC9181916; doi:10.3390/nano12111790)
Supplement: Supplementary file 1 [file nanomaterials-12-01790-s001.zip › Supplementary_materials.pdf]

Supplementary Materials: Revisiting the van der Waals Epitaxy in the Case of $(Bi_{0.4}Sb_{0.6})_2Te_3$ Thin Films on Dissimilar Substrates

Liesbeth Mulder [1], Daan H. Wielens [1], Yorick A. Birkhölzer [1], Alexander Brinkman [1] and Omar Concepción [1,*,†]

I. Substrate treatment

A high-quality epitaxial growth is essential to study the structural and electronic properties of BST thin films. This can be obtained by ensuring that the substrates have clean surfaces and saturated dangling bonds. The three substrates used in this study all receive an *ex situ* and *in situ* substrate treatment prior to the deposition process.

Ex situ treatment

The InP (111)A substrates are cleaned by an ultrasonic immersion in acetone of the as-received substrates for about 5 min, followed by an ultrasonic submersion in ethanol for 5 min. All substrates are visually inspected to verify their cleanliness using an optical microscope. If required, the substrates undergo an additional cleaning step by means of a lens tissue and isopropanol.

The Al_2O_3 (001) substrates require an additional heat treatment to create a terraced surface. Before employing the heat treatment, the Al_2O_3 substrates are cleaned using the same cleaning procedure as for the InP substrates. Subsequently, the Al_2O_3 substrates are placed in a quartz tube and heat treated in a tube furnace, at 1030°C, for 1.5 h. The typical substrate miscut of 0.33° results in terraces width of approximately 60 nm.

An equivalent cleaning procedure is applied to the $SrTiO_3$ (111) substrates, to ensure a clean surface. For the $SrTiO_3$ substrates it is desirable to have a Ti^{4+} terminated surface, even though this surface can undergo a reconstruction. In order to etch the SrO_3^{4-} termination, the substrates are first ultrasonically immersed in demineralized water for 30 min to bind OH groups to the SrO_3^{4-} terminated parts of the substrate. Thereafter, a buffered hydrofluoric acid (BHF) dip of 30 s follows to selectively etch these parts from the surface. Subsequently, the substrates are polished employing a lens tissue and isopropanol, to retrieve a clean surface. This cleaning step is succeeded by a heat treatment, in the quartz tube furnace at 950°C for 1.5 h, while supplying an oxygen flow of 150 ml/min to prevent the creation of oxygen vacancies [1]. The treated substrates from this study typically exhibit terraces with a width of approximately 130 nm.

All Al_2O_3 and $SrTiO_3$ substrates are inspected by AFM before loading into the MBE, to ensure a proper terraced surface.

In situ treatment

Alike on the Si (111) surface, dangling bonds are present on the InP (111)A surface, as is a surface oxide layer. It has previously been reported that the passivation of these dangling bonds is achieved using a strained atomic layer of Te, which allows for the growth of a relaxed BST film on Si (111) via van der Waals epitaxy [2–4]. The same passivation procedure has led to the successful growth of epitaxial Bi_2Se_3 films on InP (111)A [5]. By *in situ* annealing at 510 °C for 15 min, we achieve the deoxidation and Te passivation of the InP substrates. The high temperatures required to desorb the oxide layer, also lead to phosphorous outgassing, which is compensated for by introducing a chalcogenide overpressure, which will in turn passivate the dangling bonds on the surface [5–7]. For Al_2O_3 and $SrTiO_3$, an *in situ* pre-anneal of 1 h at 550°C was used to remove surface

contaminations. As well as with the procedure for InP, this *in situ* pre-anneal is performed in a Te-rich environment.

II. X-ray photoelectron spectroscopy

To rule out the effect of stoichiometry variations on our comparative study, the composition of 10 nm $(Bi_{1-x}Sb_x)_2Te_3$ films was determined using XPS. Figure S1 shows the survey scan (a) and the high-resolution scans of the Te 3d, Sb 3d, and Bi 4f core level spectra measured on a 10 nm film deposited on $SrTiO_3$. Table S1 presents an overview of the XPS results of all performed measurements.
While the error in the stoichiometry as determined by XPS can be a few percent (see [8]), we used identical scan settings (energy resolution, dwell time, no. of scans, etc.) and used fixed constraints for the analysis (spin-orbit splitting, area ratio between spin-orbit peaks) to minimize the error in between the scans reported here.

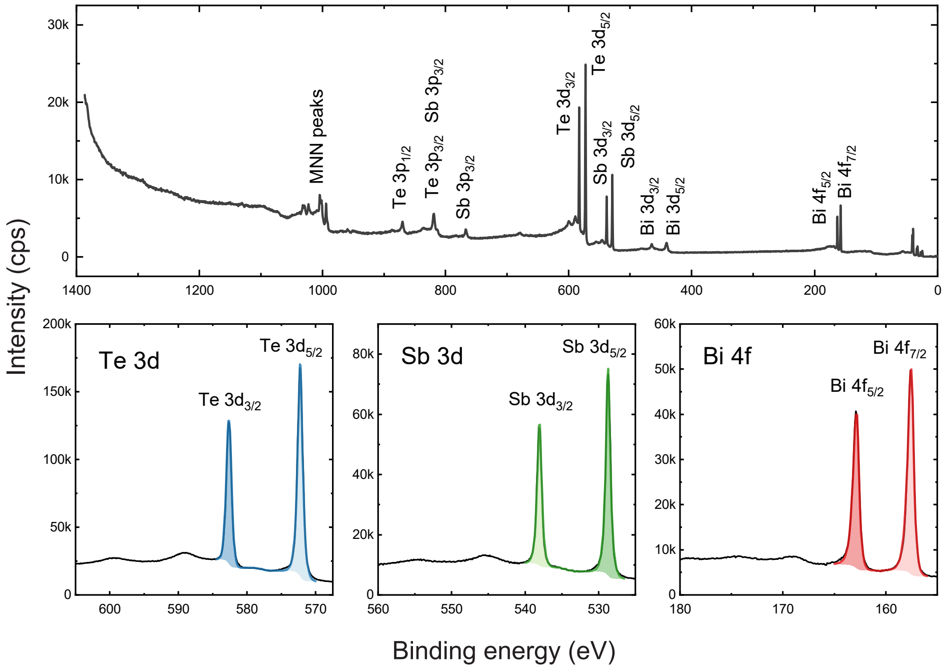

Figure S1. *In situ* XPS results for the 10 nm film deposited on $SrTiO_3$. The top panel shows the survey scan and the bottom panels show the high-resolution scans of the Te 3d, Sb 3d, and Bi 4f core level spectra used to determine the stoichiometry of the $(Bi_{1-x}Sb_x)_2Te_3$ film.

Table S1. Overview of the XPS results, including the element concentrations and determined stoichiometry, for three 10 nm $(Bi_{1-x}Sb_x)_2Te_3$ films.

Substrate	Bi (%)	Sb (%)	Te (%)	Stoichiometry
InP (111)	17.7	24.3	58.0	$(Bi_{0.42}Sb_{0.58})_2Te_3$
Al_2O_3 (001)	16.6	25.4	57.9	$(Bi_{0.40}Sb_{0.60})_2Te_3$
$SrTiO_3$ (111)	17.6	24.8	57.6	$(Bi_{0.42}Sb_{0.58})_2Te_3$

III. X-ray reflectivity analysis

The thickness of the films was determined via X-ray reflectivity (XRR). The measurements were performed using the Bruker D8 Discover diffractometer. We used various analysis methods (Fourier analysis, fitting with reflectivity software, comparison with Laue fringes) to extract the thicknesses from the measured data. The scans for the three films with a nominal thickness of 10 nm on the different substrates are shown in Figure S2.

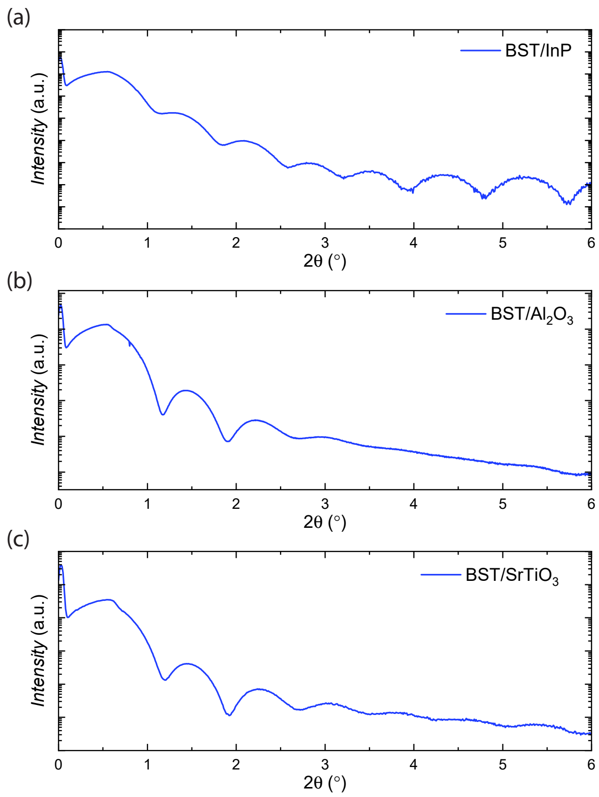

Figure S2. XRR scans of 10 nm $(Bi_{0.4}Sb_{0.6})_2Te_3$ deposited on (a) InP, (b) Al_2O_3 and (c) $SrTiO_3$.

IV. Height profiles atomic force microscopy images

Figure S3(a) and (b) reveal a height profile extracted from AFM images presented in the main text in Figure 3(d) and (f), respectively. These were the 5 nm $(Bi_{0.4}Sb_{0.6})_2Te_3$ deposited on InP (a) and $SrTiO_3$ (b). As described in the main text, some of the steps correspond to the height of a single QL, and others seem to correspond to individual substrate steps, which are 3.38 Å and 2.25 Å for InP (111)A and $SrTiO_3$ (111), respectively.

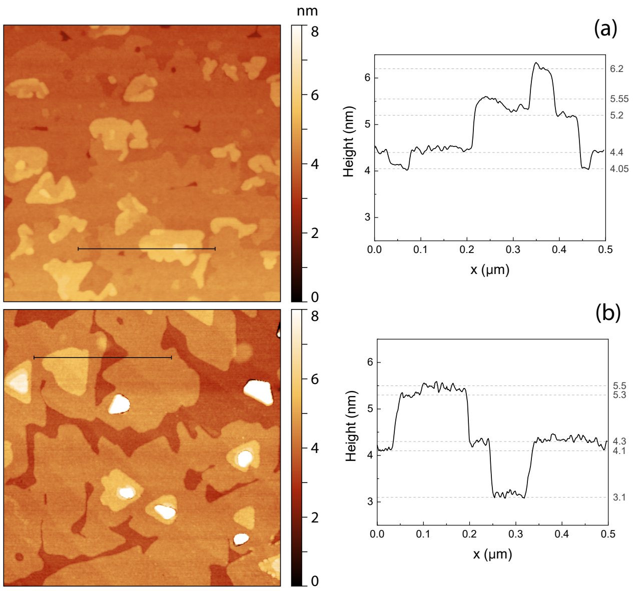

Figure S3. Height profiles extracted from AFM images of 5 nm $(Bi_{0.4}Sb_{0.6})_2Te_3$ deposited on InP (a) and $SrTiO_3$ (b), presented in the main text in Figure 3(d) and (f), respectively.

V. Phase diagrams of atomic force microscopy images

Figure S4 shows the morphology and phase data for two samples (a-b: 5 nm film on Al_2O_3; c-d: 10 nm film on $SrTiO_3$). The 5 nm film on Al_2O_3 shows some voids, which is also visible in the phase data, where a different phase is observed for these voids (e.g. top-right corner). The defects show minor changes of the phase around their corners. For the 10 nm film on $SrTiO_3$, we only observe phase differences at the edges of defects, and thus deduct that no voids towards the substrate surface are present in these films.

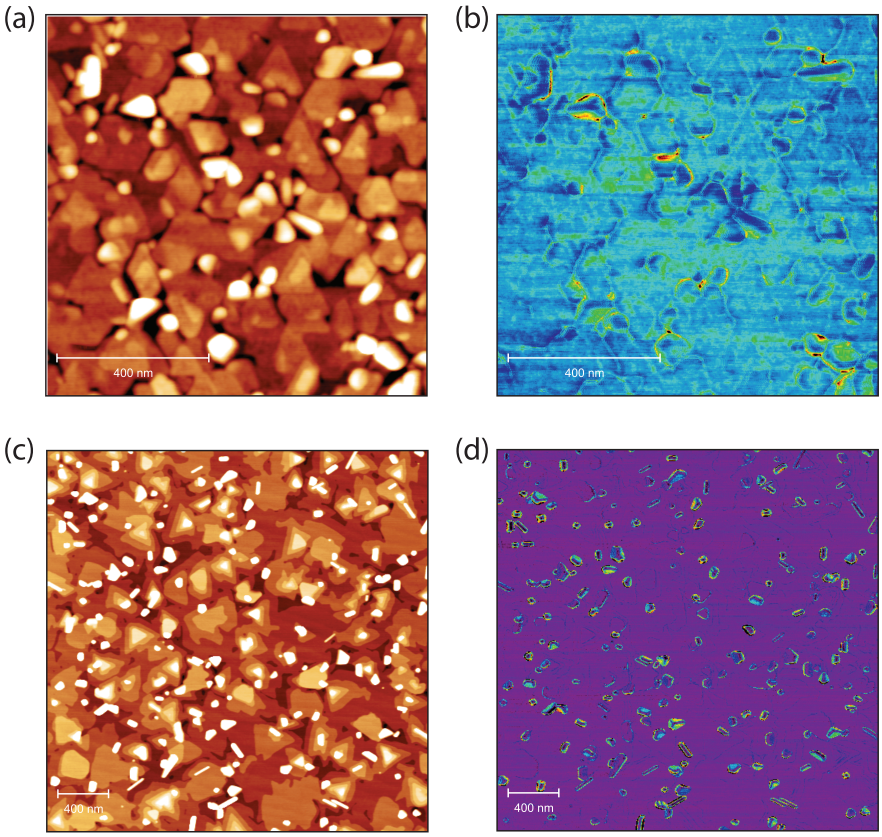

Figure S4. (a) Morphology of the 5 nm BST film on Al_2O_3 (same image as presented in the main text, Figure. 3(e)). (b) Phase data corresponding to the AFM image of panel (a). The voids to the substrate (e.g. visible in the top-right corner) have a clear difference in phase when compared with the film. The defects show minor phase differences around their edges. (c) Morphology of the 10 nm BST film on $SrTiO_3$ (same as main text, Figure 3(i)). (d) Phase data corresponding to the AFM image of panel (d). We observe no voids, but only phase differences around the edges of the defects.

VI. Scanning electron microscopy analysis

In Figure S5 we present the results of the EBSD measurements. EBSD has been used to perform a quantitative microstructural analysis on three 10 nm $(Bi_{0.4}Sb_{0.6})_2Te_3$ films, deposited on (a) InP, (b) Al_2O_3, and (c) $SrTiO_3$ substrates. The samples are mounted on the sample holder by means of silver epoxy, and an additional droplet of silver epoxy on the corner of the sample is used to make contact between the film and sample holder to prevent charging effects.

The SEM images on the left reveal the morphology of the films. The inverse pole figure (IPF) data shows the orientation of the film with respect to the Z (out-of-plane) or X (in-plane) axis. The inset in the IPF Z image of panel (a) shows the color mapping used in the IPF figures. The IPF Z images show that the films are well-oriented in the (001) direction, which corresponds to the results from the symmetric XRD diffractograms, see

Figure 1 in the main text. We attribute the relatively low measurement yield on Al_2O_3 and $SrTiO_3$ to the randomly inclined crystals present in the $(Bi_{0.4}Sb_{0.6})_2Te_3$ films on these substrates.

The IPF X images provide information on the in-plane orientation of the films, which present similar results to the pole figures in the main text. Here, we see a clear difference between the different substrates. The film on InP shows two domains, in stark contrast to the film on Al_2O_3 featuring almost a single phase. The film grown on $SrTiO_3$ exhibits a lot of different domains (although mostly oriented along two preferred directions).

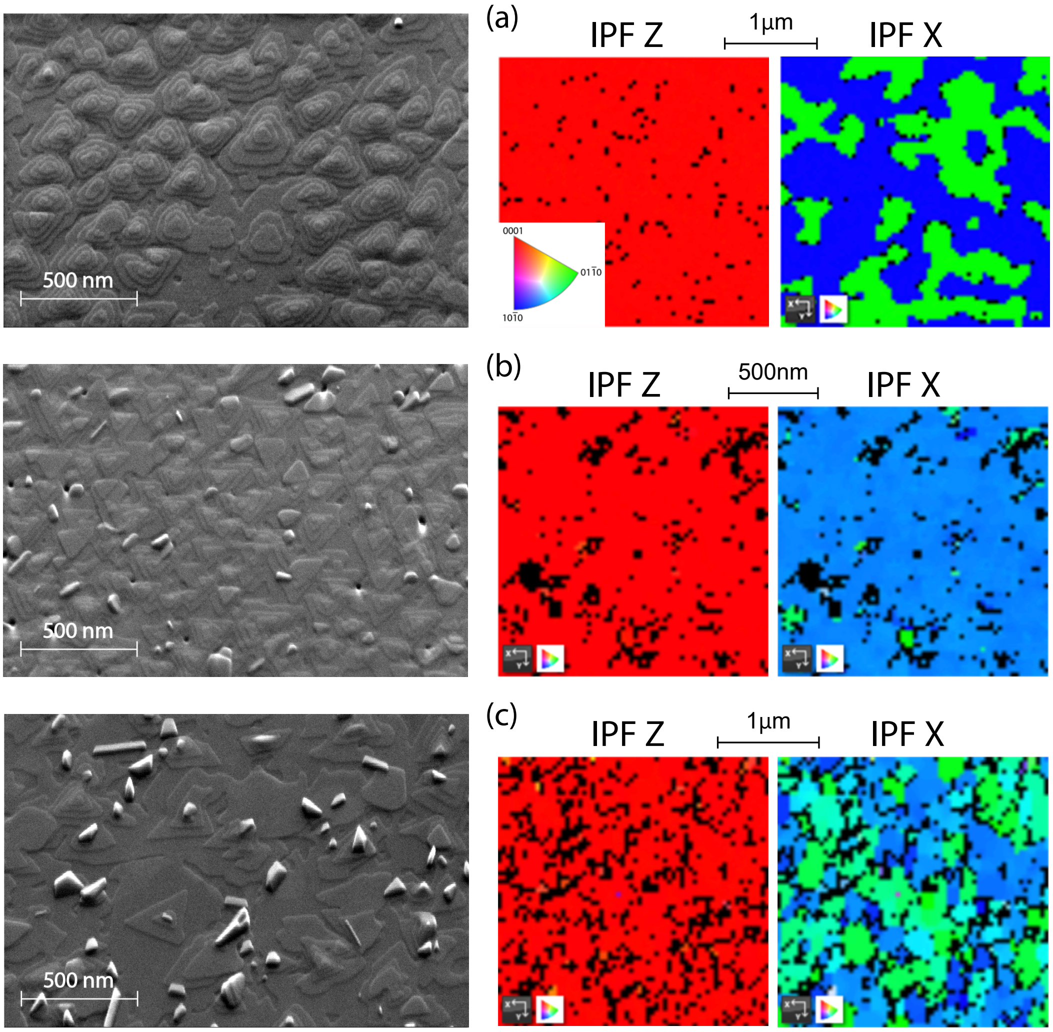

Figure S5. EBSD results for the three 10 nm films deposited on (a) InP, (b) Al_2O_3, and (c) $SrTiO_3$ substrates. The SEM images on the left show the morphology of the films, and the IPF panels show the crystal directions parallel to the respective axis (Z or X). The inset in the IPF Z image of panel (a) shows the color mapping used in the IPF images.

VII. Pole figures substrates

In Figure S6 we show pole figures of both the films (top row) and their corresponding substrates (bottom row). From the figures, it is clear that the azimuthal angles of the spots of the BST films coincide very well with those of the substrates.

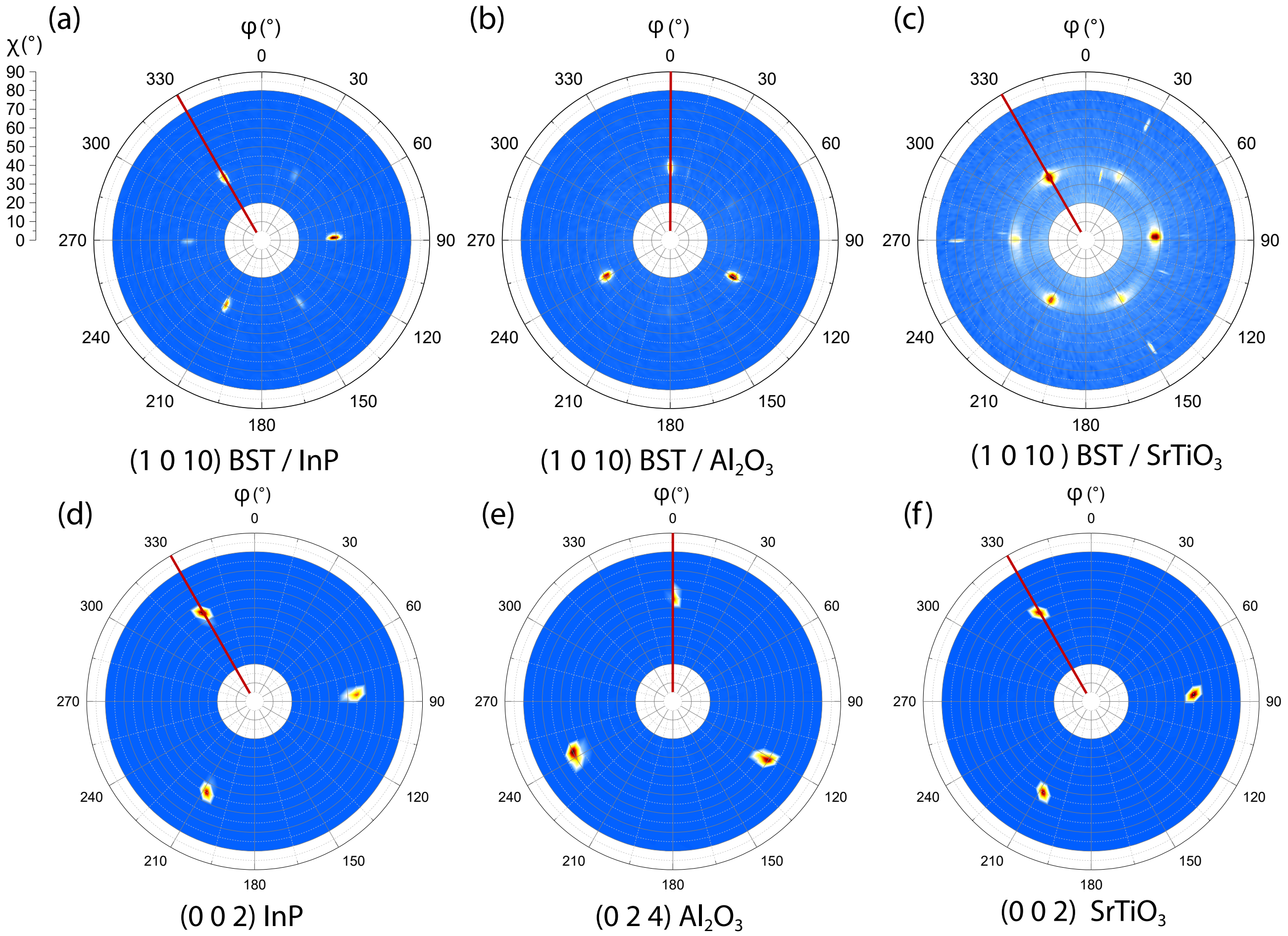

Figure S6. Pole figures taken in symmetric $2\theta - \omega$ configuration on 10 nm BST films deposited on InP (111)A, Al_2O_3 (001), and $SrTiO_3$ (111). φ and χ present the azimuthal and tilt angle, respectively. The pole figures presented in (a)-(c) map the BST {10$\underline{10}$}, whereas (d)-(f) map the corresponding InP {002}, Al_2O_3 {024} and $SrTiO_3$ {002}. The azimuthal angles of the spots of the BST films coincide with those of the substrates.

VIII. Pole figures films - different representation

In Figure S7 we plot the pole figures of the main text, Figure 4, in a different manner. From these plots, it can be deduced that the ratio between the two domains is very large for Al_2O_3 and somewhat smaller for InP, which is also consistent with the EBSD analysis in Figure S5.

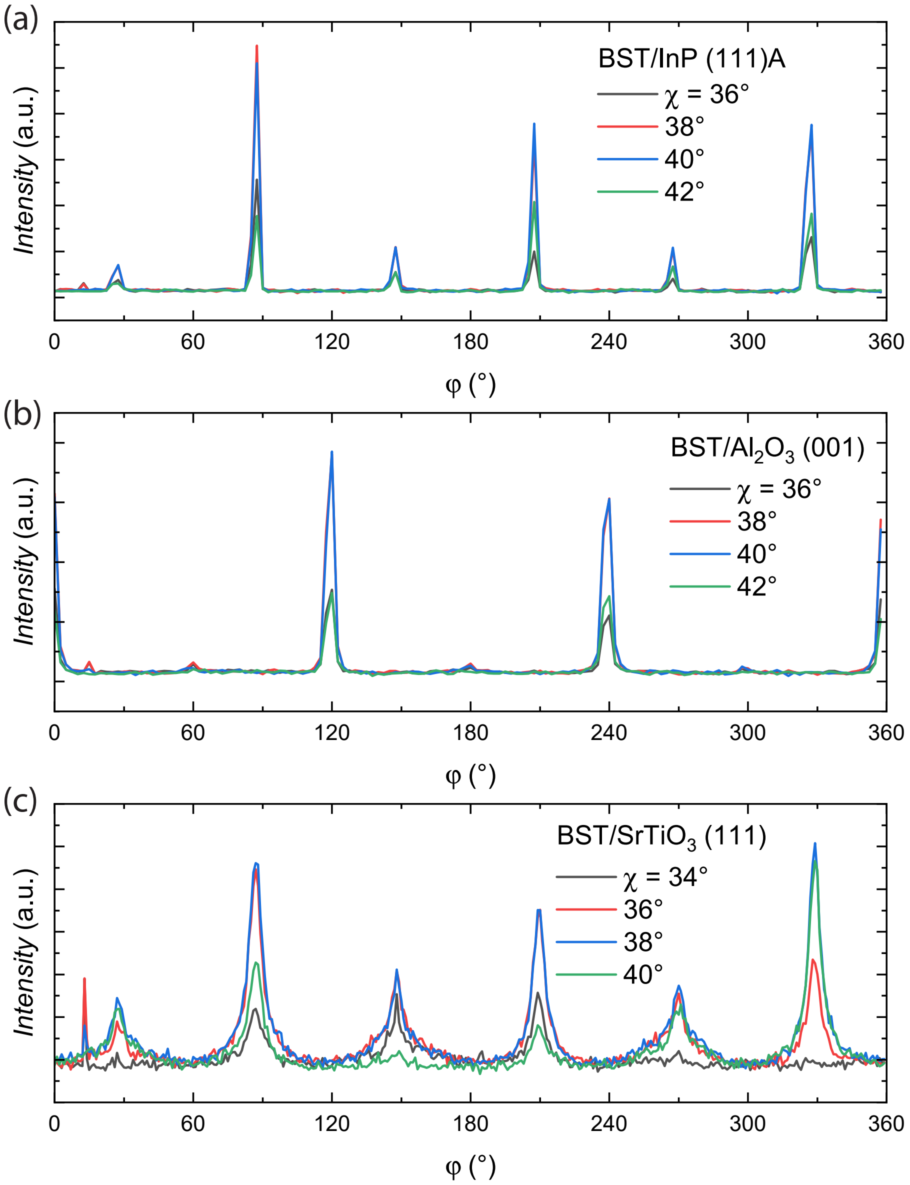

Figure S7. Phi-scan representation of the pole figures that are shown in the main text, Figure 4.

IX. Calculations of rotational alignment film and substrates

The calculations visualized in Figure S8 are performed using the WinWulff Stereographic software. The calculations performed on BST and InP, presented in Figure S8(a), reveal that the InP (133) reflection (black dots) can be found at the same azimuthal angle as the $(Bi_{1-x}Sb_x)_2Te_3$ (01$\underline{20}$) reflection (red dots), and exhibits a 60° rotation with the $(Bi_{1-x}Sb_x)_2Te_3$ (10$\underline{19}$) reflection (blue dots). In contrast, the calculations show that the Al_2O_3 (018) (b) and $SrTiO_3$ (112) (c) reflections can be found at the same azimuthal angle as the $(Bi_{1-x}Sb_x)_2Te_3$ (10$\underline{19}$) reflection.

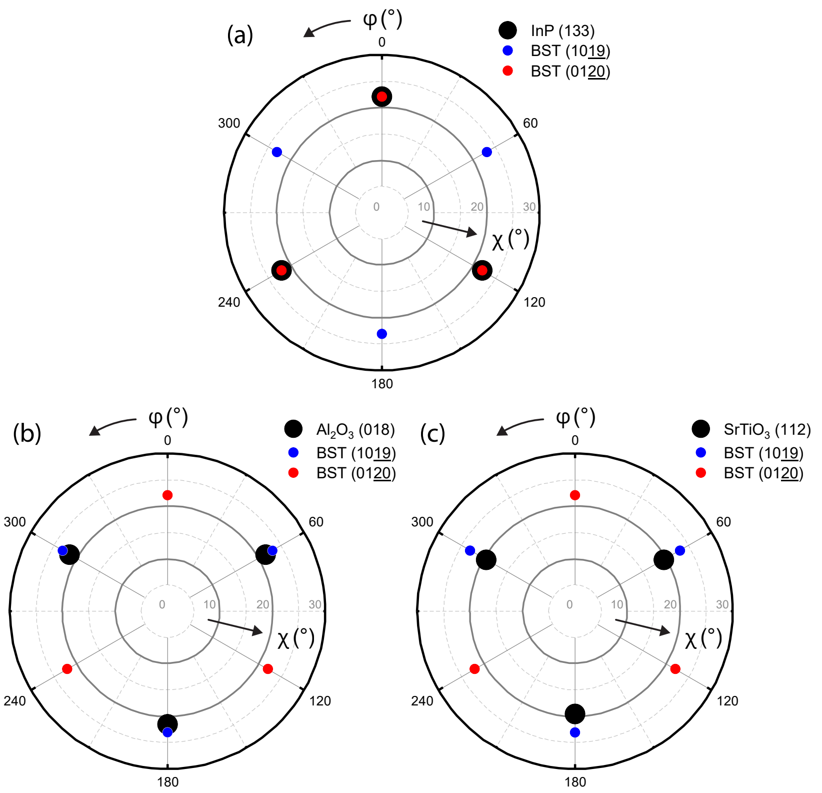

Figure S8. Calculations of relative azimuthal angles, φ, for the $(Bi_{1-x}Sb_x)_2Te_3$ (01$\underline{20}$) and (10$\underline{19}$), the (a) InP (133), (b) Al_2O_3 (018), and (c) $SrTiO_3$ (112) reflections, for which corresponding RSMs are presented in Figure 2 in the main text. φ and χ present the azimuthal and tilt angle, respectively.

X. Elemental map acquired using EDX on $(Bi_{0.4}Sb_{0.6})_2Te_3$ on Al_2O_3

In the main text, elemental maps acquired using EDX are presented on a 10nm $(Bi_{0.4}Sb_{0.6})_2Te_3$ film deposited on $SrTiO_3$, which reveal the accumulation of Sb near the substrate/film interface. The elemental maps presented in Figure S9 reveal the same effect in $(Bi_{0.4}Sb_{0.6})_2Te_3$ film deposited on Al_2O_3.

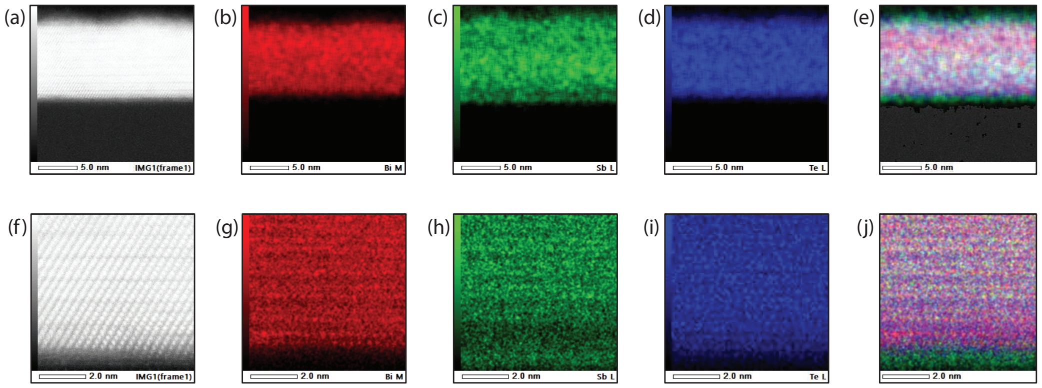

Figure S9. Elemental maps acquired using EDX on a 10nm $(Bi_{0.4}Sb_{0.6})_2Te_3$ film deposited on Al_2O_3. (a)-(e) reveal both the substrate/film interface as the top surface of the BST film, whereas (f)-(j) focus on the substrate/film interface area. The EDX analysis reveals the accumulation of Sb near both film interfaces. (a) and (f) present a reference image of the area on which the EDX mapping is performed. (b)-(d) and (g)-(i) show the individual elemental maps of Bi (red), Sb (green) and Te (blue), and (e) and (j) present an overlay of elemental maps. The analysis reveals the ordering of Sb in the interfacial layer.

References

1. Koster, G.; Kropman, B.L.; Rijnders, G.J.H.M.; Blank, D.H.A.; Rogalla, H. Quasi-ideal strontium titanate crystal surfaces through formation of strontium hydroxide. *Appl. Phys. Lett.* **1998**, *73*, 2920–2922.
2. Borisova, S.; Krumrain, J.; Luysberg, M.; Mussler, G.; Grützmacher, D. Mode of growth of ultrathin topological insulator Bi_2Te_3 films on Si (111) substrates. *Cryst. Growth Des.* **2012**, *12*, 6098–6103.
3. Kampmeier, J.; Borisova, S.; Plucinski, L.; Luysberg, M.; Mussler, G.; Grützmacher, D. Suppressing twin domains in molecular beam epitaxy grown Bi_2Te_3 topological insulator thin films. *Cryst. Growth Des.* **2015**, *15*, 390–394.
4. Mussler, G. Molecular-beam epitaxy of 3D topological insulator thin films and devices on Si substrates. *Phys. Status Solidi B* **2021**, *258*, 2000007.
5. Guo, X.; Xu, Z.J.; Liu, H.C.; Zhao, B.; Dai, X.Q.; He, H.T.; Wang, J.N.; Liu, H.J.; Ho, W.K.; Xie, M.H. Single domain Bi_2Se_3 films grown on InP(111)A by molecular-beam epitaxy. *Appl. Phys. Lett.* **2013**, *102*, 151604.
6. Tarakina, N.V.; Schreyeck, S.; Luysberg, M.; Grauer, S.; Schumacher, C.; Karczewski, G.; Brunner, K.; Gould, C.; Buhmann, H.; Dunin-Borkowski, R.E.; et al. Suppressing twin formation in Bi_2Se_3 thin films. *Adv. Mater. Interfaces* **2014**, *1*, 1400134.
7. Ginley, T.P.; Wang, Y.; Law, S. Topological insulator film growth by molecular beam epitaxy: A review. *Crystals* **2016**, *6*, 154.
8. Bouwmeester, R.L.; de Hond, K.; Gauquelin, N.; Verbeeck, J.; Koster, G.; Brinkman, A. Stabilization of the Perovskite Phase in the Y–Bi–O System By Using a BaBiO3 Buffer Layer. *Phys. Status Solidi (RRL)—Rapid Res. Lett.* **2019**, *13*, 1800679.
